# New Insights into the Biological Functions of Essential TsaB/YeaZ Protein in *Staphylococcus aureus*

**DOI:** 10.3390/antibiotics13050393

**Published:** 2024-04-25

**Authors:** Haiyong Guo, Ting Lei, Junshu Yang, Yue Wang, Yifan Wang, Yinduo Ji

**Affiliations:** 1 School of Life Science, Jilin Normal University, Siping 136000, China; guohaiyong78@jlnu.edu.cn (H.G.);; 2 Department of Veterinary and Biomedical Sciences, College of Veterinary Medicine, University of Minnesota, Saint Paul, MN 55108, USA; leiting@cpgroup.cn (T.L.);

**Keywords:** *Staphylococcus aureus*, TsaB/YeaZ, autolysis, transcriptional regulator, RNAseq

## Abstract

TsaB/YeaZ represents a promising target for novel antibacterial agents due to its indispensable role in bacterial survival, high conservation within bacterial species, and absence of eukaryotic homologs. Previous studies have elucidated the role of the essential staphylococcal protein, TsaB/YeaZ, in binding DNA to mediate the transcription of the *ilv*-*leu* operon, responsible for encoding key enzymes involved in the biosynthesis of branched-chain amino acids—namely isoleucine, leucine, and valine (ILV). However, the regulation of ILV biosynthesis does not account for the essentiality of TsaB/YeaZ for bacterial growth. In this study, we investigated the impact of TsaB/YeaZ depletion on bacterial morphology and gene expression profiles using electron microscopy and deep transcriptomic analysis, respectively. Our results revealed significant alterations in bacterial size and surface smoothness upon TsaB/YeaZ depletion. Furthermore, we pinpointed specific genes and enriched biological pathways significantly affected by TsaB/YeaZ during the early and middle exponential phases and early stationary phases of growth. Crucially, our research uncovered a regulatory role for TsaB/YeaZ in bacterial autolysis. These discoveries offer fresh insights into the multifaceted biological functions of TsaB/YeaZ within *S. aureus*.

## 1. Introduction

*Staphylococcus aureus* is one of the major human pathogens that can cause a variety of superficial and systematic infections. The multidrug-resistant *S. aureus* isolates, especially methicillin-resistant *S. aureus* (MRSA), have caused serious public health concerns due to the limitation of treatment options [1,2]. Therefore, there is an urgent need for the development of alternative strategies to combat multidrug-resistant *S. aureus* infections.

TsaB/YeaZ is a promising target for novel antibacterial agents because it is necessary for bacterial survival. It is highly conserved within *S. aureus*. Furthermore, the TsaB/YeaZ orthologs are required for growth in many bacteria, including *E. coli* [3], *Pseudomonas aeruginosa* [4,5], *Streptococcus pneumoniae* [6], and *S. aureus* [7]. Importantly, there are no TsaB/YeaZ orthologs in any eukaryotic cells, indicating the high selectivity of TsaB/YeaZ inhibitors. Therefore, TsaB/YeaZ is a promising target for the development of novel broad-spectrum antibacterial agents.

TsaB/YeaZ possesses multiple functions in various bacteria. It is well documented that TsaB is involved in the post-transcriptional modification of N6-threonylcarbamoyl adenosine (t^6^A) biosynthesis [8,9,10] and is distinctively dispensable in *Streptococcus mutans* [11]. In *S. aureus*, the *tsaB/yeaZ* gene is located on an essential *tsaD/gcp* operon composed of *sa1857*, *sa1856 (tsaB/yeaZ)*, *sa1855*, and *sa1854 (tsaD/gcp)* genes that are co-transcribed [12,13]. Staphylococcal TsaB/YeaZ and TsaD/Gcp are also involved in t^6^A biosynthesis [7], and the interaction of TsaB/YeaZ with TsaD/Gcp contributes to the essentiality of TsaD/Gcp [14]. We have demonstrated that staphylococcal TsaD/YeaZ binds the promoter region of the *ilv-leu* operon and negatively regulates the transcription of *ilv-leu* operon responsible for the biosynthesis of the branched-chain amino acids (BCAAs) in *S. aureus*, indicating its DNA-binding ability [7]. Both *E. coli* and *Salmonella typhimurium* TsaB/YeaZ can bind to the other two essential proteins, YjeE and TsaD/YgiD [15,16]. TsaB/YeaZ, of *E. coli* and *Vibrio Harveyi*, exhibits the protease activity [16,17,18] and specifically cleaves TsaD/YgjD [16]. In contrast, both *S. aureus* and *S. typhimurium* TsaB/YeaZ lacks protease activity to cleave TsaD/Gcp [14,15]. These discrepancies suggest that the essentiality of TsaB/YeaZ may be not attributable to the protease activity of *E. coli* TsaB/YeaZ. Moreover, structural analysis of YeaZ homologs from various bacterial species has indicated that YeaZ exhibits a classic actin-like nucleotide-binding fold and belongs to the acetate and sugar kinase/Hsc70/actin (ASKHA) superfamily [16,17,19,20]. *S. typhimurium* YeaZ interacts with another essential protein, YgjD, and YjeE to form a ternary complex [21]. In addition, *S. typhimurium* YeaZ functions as a resuscitation-promoting factor to allow bacteria to go from being viable but non-culturable to being culturable [18,22,23,24,25]. Taken together, the above data indicate that the TsaB/YeaZ homologs possess distinct biological functions in different bacterial species.

Our previous studies revealed that the essentiality of TsaB/YeaZ is not attributable to its negative regulation of the *ilv-leu* operon in *S. aureus* [7]. To elucidate the essential functions of TsaB/YeaZ, in this study, we determined the impact of TsaB/YeaZ on bacterial morphology using electronic microscopy and their influences on bacterial autolysis. Furthermore, we performed the kinetic transcriptomic studies, identified biological pathways that are involved in *S. aureus* growth and bacterial autolysis, and revealed that TsaB/YeaZ mediates similar biosynthesis pathways during different growth phases. Furthermore, we revealed the protein networks that interact with TsaB/YeaZ. Our results provide new insights into the essential functions of TsaB/YeaZ in *S. aureus*.

## 2. Results

### 2.1. The Deletion of TsaB/YeaZ Leads to Unusual Cellular Morphologies

**Figure 1:**
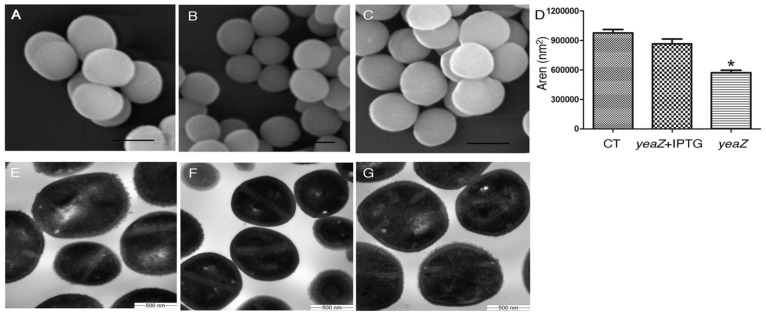
Scanning and transmission electron micrographs of *S. aureus* with TsaB/YeaZ depletion. SEM was taken on the control JW290011 (**A**), *tsaB*/*yeaZ* conditional knockout strain JW290211, grown in TSB without IPTG (**B**), and with 100 µM IPTG (**C**). The scale bar in SEM represents 1 µm. The bacterial cell size was measured as the area of cells under SEM (**D**). The cell size of parental strain JW290111 was measured as a control. Error bars represent standard errors or the means; *n* = 20. Star means the statistical difference, *p* < 0.05. TEM was taken on the control JW290011 (**E**), YeaZ conditional knockout strain JW290211, grown in TSB without IPTG (**F**), and with 100 µM IPTG (**G**).

Our previous studies have demonstrated the essentiality of TsaB/YeaZ for *S. aureus* growth [7]. To pinpoint the mechanisms of TsaB/YeaZ in mediating bacterial growth, we determined the impact of TsaB/YeaZ on bacterial morphology using scanning electron microscopy (SEM) and transmission electron microscopy (TEM). Compared with the control strain (Figure 1A), the depletion of TsaB/YeaZ obviously decreased the cell size of *tsaB*/*yeaZ* conditional knockout strain JW290411 (Figure 1B). However, the addition of 100 μM of IPTG to induce the expression of TsaB/YeaZ restored the cell size of the *tsaB*/*yeaZ* conditional knockout mutant (Figure 1C). To quantitatively examine the effect of TsaB/YeaZ on bacterial cell size, we randomly picked 20 single cells to measure the cell area using iTem software. The cell size of the TsaB/YeaZ depleted strain decreased 1.5-fold compared with that of the wildtype control, from 9.47 × 10^5^ nm^2^ to 6.10 × 10^5^ nm^2^ (Figure 1D). Moreover, the transmission electron micrographs of *S. aureus* showed that most of the TsaB/YeaZ-depleted bacterial cells lost their rusting appearance and exhibited a smooth cell surface (Figure 1F) compared with the parental control (Figure 1E). Moreover, the addition of IPTG to induce the expression of TsaB/YeaZ restored the morphology of the cell wall surface of *S. aureus* (Figure 1G).

### 2.2. Identification of Genes That Are Differentially Expressed during the Downregulation of TsaB/YeaZ

**Figure 2:**
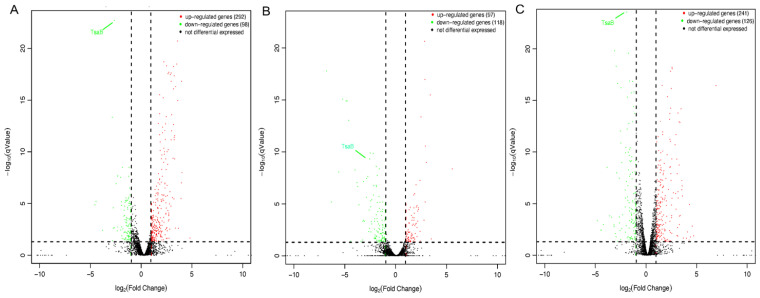
Volcano plot of the differentially expressed genes during the depletion of TsaB/YeaZ. (**A**) at the early log phase of growth (OD600nm = ~0.2); (**B**) at the middle log phase of growth (OD600nm = ~0.5; (**C**) at the early stationary phase of growth (OD600nm = ~1.0). The y-axis shows the −log10 *p*-value for each mRNA, while the x-axis shows the log2 fold change for that mRNA relative to controls. Red dots indicate upregulated genes, green dots indicate downregulated genes, and black dots indicate non-significantly differentially expressed genes between the uninduced and induced (with IPTG) group of *tsaB*/*yeaZ* conditional knockout mutant.

To elucidate the essential mechanisms of TsaB/YeaZ involved in *S. aureus* growth and bacterial cell morphology, we examined the impact of TsaB/YeaZ on the transcriptomes of *S. aureus* during different stages of growth by using RNAseq analysis. We conducted DESeq analysis to identify genes whose transcriptions were significantly different at q Value ≤ 0.05 and log2 Fold Change ≥ 1. Six groups were included and analyzed at every growth phase of culture between, both in the absence and in the presence of inducer IPTG (100 μM). In the control group, no significant difference in gene transcriptions was identified between the absence and presence of 100 μM IPTG at the middle log (OD600 = 0.5) and early stationary (OD600 = 1.0) phases of growth; two genes were upregulated, and three genes were downregulated at the early log (OD600 = 0.2) phase of growth after adding 100 μM IPTG, indicating the inducer IPTG had limited or negatable influence on the transcriptome of *S. aureus*. However, the downregulation of TsaB/YeaZ in the absence of IPTG significantly altered 390, 215, and 367 genes’ transcription at the early and middle log and early stationary phases of growth, respectively (Figure 2, Tables S1–S3 and S5).

Interestingly, we found that in the early log phase of growth, the depletion of TsaB/YeaZ remarkably decreased the transcription of *dltC* encoding a D-alanine-poly(phosphoribitol) ligase subunit, which is essential for bacterial growth [26,27], which may contribute to the function of TsaB/YeaZ on bacterial growth (Table S1). On the other hand, the depletion of TsaB/YeaZ dramatically increased the transcription of the *ssb* gene, encoding a single-strand DNA-binding protein (SSB), and *capABCDEFGLMN* operon, responsible for the capsule polysaccharide biosynthesis from the early log to early stationary phases of growth (Tables S1–S3). Moreover, the depletion of TsaB/YeaZ significantly enhanced the transcription of *ilv*-*leu* operons (*ilvABCD*-*leuBCD*), responsible for the biosynthesis of branched-chain amino acids [7].

**Figure 3:**
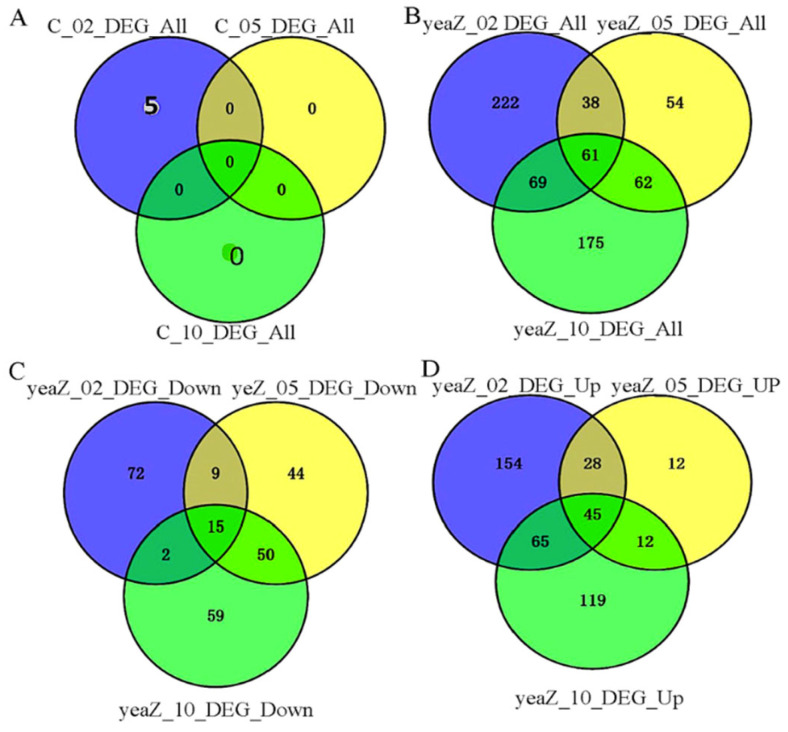
The diagram illustrates the overlapped differentially expressed genes at various growth phases during the downregulation of TsaB/YeaZ. (**A**) Total differentially expressed genes for the control strain JW29011 (**C**) across different growth phases without and with 100 μM IPTG; (**B**) Total differentially expressed genes for the IPTG-induced *tsaB*/*yeaZ* expression mutant JW290211 (yeaZ) across different growth phases in the absence and presence of 100 μM IPTG; (**C**) The differentially downregulated genes for the IPTG-induced *tsaB*/*yeaZ* expression mutant JW290211(yeaZ) across different growth phases in the absence and presence of 100 μM IPTG; (**D**) The differentially upregulated genes for the IPTG-induced *tsaB*/*yeaZ* expression mutant JW290211 (yeaZ) between the absence and presence of 100 μM IPTG. C_02, C_05, and C_10 represent the growth of the control strain JW29011 in TSB without IPTG at OD600nm ≈ 0.2, 0.5, and 1.0, respectively; yeaZ_02, yeaZ_05, and yeaZ_10 represent the growth of the IPTG-induced *tsaB*/*yeaZ* expression mutant in TSB without IPTG at OD600nm ≈ 0.2, 0.5, and 1.0, respectively.

To further characterize the genes whose expressions are significantly affected by TsaB/YeaZ, we used the VENN2.1 tool to integrate the three sets of differentially expressed genes at different phases of growth. We found only 5 integrations in the 3 sets of differentially expressed genes in wildtype control, including E5491_RS01135, _RS13875, _RS08970, _RS05080, and _RS06615, all at the early log phase of growth, and no overlap of differentially expressed genes appeared among different phases of growth for the control strain (Figure 3). In contrast, 15 significantly downregulated and 45 significantly upregulated genes overlapped, respectively, among different phases of growth during the depletion of TsaB/YeaZ (Figure 3, see details in Table S4). Moreover, the differentially expressed genes downregulated by TsaB/YeaZ had no overlap with those of the wildtype control, suggesting the selective impact of TsaB/YeaZ on the transcriptomes of *S. aureus*.

### 2.3. The Depletion of TsaB/YeaZ Alters Bacterial Autolysis and Cell Wall Recycling

**Figure 4:**
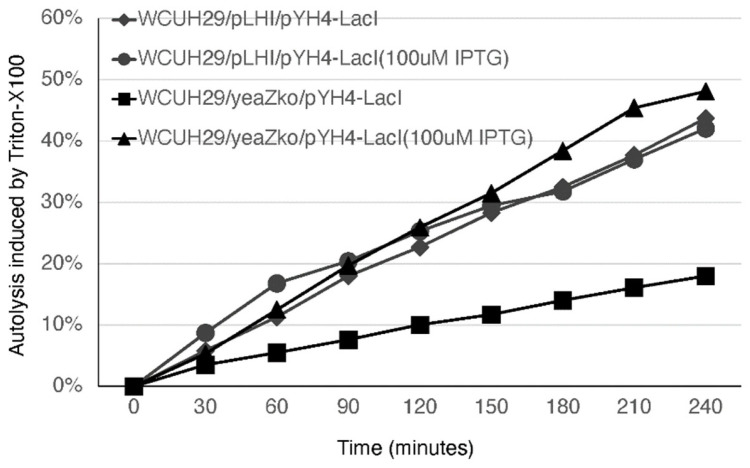
Effect of depleted TsaB/YeaZ on Triton X-100-induced autolysis of *S. aureus*. The *Pspac*-regulated *tsaB/yeaZ* mutant strain, WCUH29/YKO/pYH4-lacI, and the control, WCUH29/pLH/pYH4, were grown in TSB-NaCl in the presence and absence of IPTG (100 μM). Results were normalized to the OD_580_ at time zero (OD_0_). The percent lysis was determined as follows: percent lysis at time *t* = [(OD_0_ − OD*_t_*)/OD_0_] × 100. The experiments were repeated at least three times. The figure represents the results of one experiment.

Our transcriptomic analysis showed that depleting TsaB/YeaZ significantly downregulated the transcription of *murQ*, *cidA*, and *lrgA* genes in the middle log phase of growth (Table S2), which was further confirmed by using qPCR analysis (Table 1). MurNAc-6P etherase is encoded by *murQ* and converts intracellular MurNAc-6P to N-acetylglucosamine-6-phosphate and d-lactate [28]. MurQ is involved in bacterial peptidoglycan recycling [28]. CidA and LrgA proteins function as holins to mediate bacterial autolysis of *S. aureus* [29,30]. Therefore, we hypothesized that TsaB/YeaZ is involved in autolysis by mediating the expression of CidA and LrgA. To test it, we conducted autolysis assays with detergent Triton-X-100. The addition of IPTG had no influence on the autolysis for the control strain; in contrast, the depletion of TsaB/YeaZ in the absence of IPTG dramatically decreased the bacterial autolysis (Figure 4). Moreover, the addition of 100 μM IPTG to induce the expression of TsaB/YeaZ restored the autolysis capacity of the *tsaB*/*yeaZ* knockout strain to a similar level to the controls (Figure 4).

To elucidate the potential mechanism through which TsaB/YeaZ participates in bacterial autolysis, we assessed its impact on the expression of autolysis-associated genes in *S. aureus*, namely, *cidA*, *atl*, *lytM*, *lytN*, and *femA*, utilizing qPCR analysis. Downregulation of *tsaB*/*yeaZ* by approximately 2-fold, using the *Pspac*-regulated *tsaB*/*yeaZ* expression strain, resulted in a noteworthy 2-fold reduction in the expression of *cidA*, *femA*, and *lytM* genes. Interestingly, *lytN* and *atl* exhibited a more substantial decrease of over 2-fold (Table 1). No apparent alterations in the expression of these genes were observed in the control strain.

### 2.4. The Down Regulation of TsaB/YeaZ Affects the Transcriptions of Some tRNA Genes

TsaB/YeaZ plays a crucial role in tRNA modification, particularly in the biosynthesis of threonylcarbamoyl adenosine (t6A) [9,10,12,31]. This modification is integral for the selection of start codons, decoding ANN codons, and enhancing translation efficiency by preventing intramolecular base pairing between amino acids U33 and A37 [9,31]. Our RNAseq analysis uncovered that the downregulation of TsaB/YeaZ led to a decrease in the transcription of tRNA-leu, tRNA-Agr, tRNA-Asp, tRNA-Thr, and tRNA-Ser during the early log phase of growth (Table S1). In contrast, during the middle log phase of growth, the transcriptional levels of tRNA-Ilu, tRNA-Leu, and tRNA-Ile increased following the downregulation of TsaB/YeaZ (Table S2). No changes in the transcription of these tRNA genes were observed during the early stationary phase of growth after the depletion of TsaB/YeaZ (Table S3).

### 2.5. The Depletion of TsaB/YeaZ Affects the Transcriptions of Multiple Genes Encoding Virulence Factors

Interestingly, our transcriptomic analysis revealed that the depletion of TsaB/YeaZ significantly downregulated the transcriptions of virulence genes *spa* (encoding protein A) and *pmtD* (encoding a phenol-soluble modulins transporter) in the early exponential phase of growth (Table S1). In the mid-exponential and early stationary phases of growth, the depletion of TsaB/YeaZ significantly affected numerous virulence factors, including cell surface protein SasD and SasG and toxin genes such as *lukG*, *lukH*, *hly* (alpha-hemolysin), *hlgA*, *hlgB*, *hlgC*, *sasA* (glycoprotein adhesin), *splC* and *splF* (encoding serine protease), PSM-alpha-2, beta-class phenol-soluble modulin, *clpL* (ATP-dependent Clp protease), CHIPS (chemotaxis-inhibiting protein), *norB* efflux system, and *tet* (38) (see details in Tables S2 and S3). Moreover, TsaB/YeaZ mediated the transcription of virulence regulators, including staphpostatin A (protease inhibitor), *mraZ*, a multifunctional transcriptional regulator of *S. aureus* pathogenicity [32], *sarS*, *msa* (*sarA* expression modulator), and the SrrAB two-component system, which regulates staphylococcal virulence factors and affects bacterial growth under anabolic conditions [33,34].

To validate our findings, we performed qPCR analysis, which indicated that the approximately 2-fold downregulation of TsaB/YeaZ substantially reduced the expression of virulence factors LukH and HlgC, along with the two-component regulator SrrA (Table 1). These results align with the outcomes identified in our RNAseq analysis.

### 2.6. Identify the Enriched Biological Pathways That Are Significantly Affected by TsaB/YeaZ

**Figure 5:**
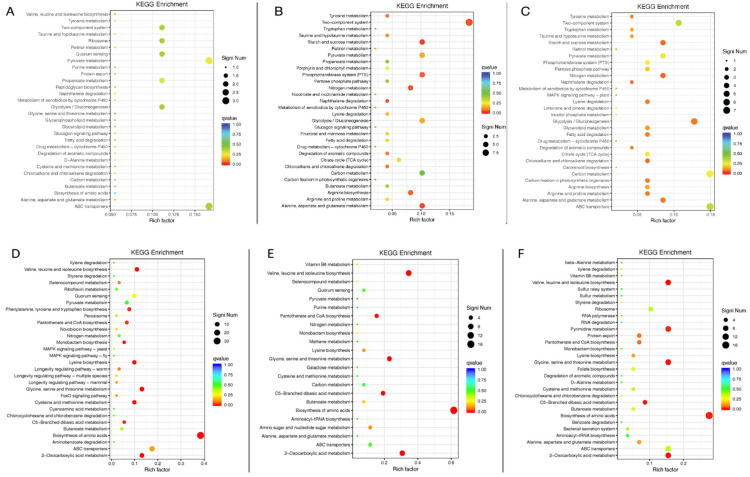
The significantly enriched pathways affected by downregulating TsaB/YeaZ in *S. aureus* in KEGG enrichment biological pathway analysis. (**A**) The significantly downregulated enrichment pathways by the depletion of TsaB/YeaZ at the early log phase of growth (OD600nm = ~0.2); (**B**) The significantly downregulated enrichment pathways by the depletion of TsaB/YeaZ at the middle log phase of growth (OD600nm = ~0.5); (**C**) The significantly downregulated enrichment pathways by the depletion of TsaB/YeaZ at the early stationary phase of growth (OD600nm = ~1.0). (**D**) The significantly upregulated enrichment pathways by the depletion of TsaB/YeaZ at the early log phase of growth (OD600nm = ~0.2); (**E**) The significantly upregulated enrichment pathways by the depletion of TsaB/YeaZ at the middle log phase of growth (OD600nm = ~0.5); (**F**) The significantly upregulated enrichment pathways by the depletion of TsaB/YeaZ at the early stationary phase of growth (OD600nm = ~1.0). The Rich factor is the ratio of the differentially expressed number of genes in the pathway and the total number of genes in the pathway. The higher the Rich factor, the higher the degree of enrichment. QValue is the p-value after the multiple hypothesis test correction, in the range of 0 to 1; the closer the QValue is to zero, the more significant the enrichment.

To determine whether the differentially expressed genes during downregulating TsaB/YeaZ are focused on certain biological functions, we performed the KEGG pathway (Figure 5) and Gene Ontology (GO) biological process (BP) (Figure S1) functional enrichment analyses. At the early log phase (OD600nm ~0.2), the significantly downregulated and enriched pathways among the 98 differentially downregulated genes affected by TsaB/YeaZ included the two-component system, ribosome, quorum sensing, pyruvate and propanoate metabolism, glycolysis, and ABC transporters (Figure 5A). In contrast, biosynthesis pathways for amino acids included branched-chained amino acids (valine, leucine, and isoleucine) biosynthesis, lysine biosynthesis, 2-oxocarboxylic acid metabolism, glycine, and serine and threonine metabolism, as well as pathways related to cysteine and methionine metabolism and some ABC transporters, and enriched pathways were significantly upregulated among the 292 differentially upregulated genes by TsaB/YeaZ (Figure 5D). At the middle log phase of growth (OD600nm ~0.5), the significantly downregulated enrichment pathways among 118 differentially downregulated genes by TsaB/YeaZ included the two-component system, arginine biosynthesis, starch and sucrose metabolism, pyruvate metabolism, phosphotransferase system (PTS), nitrogen metabolism, glycolysis, carbon metabolism, as well as alanine, aspirate, and glutamate metabolism (Figure 5B). However, biosynthesis pathways for amino acids, such as branched-chained amino acids (valine, leucine, and isoleucine) biosynthesis, pantothenate and CoA biosynthesis, 2-oxocarboxylic acid metabolism, C5-branched dibasic acid metabolism, and glycine, serine, and threonine metabolism pathways, were remarkably upregulated among the 97 differently upregulated genes by TsaB/YeaZ (Figure 5E). Similarly, at the early stationary phase of growth (OD600nm ~1.0), the significantly downregulated enrichment pathways among the 126 differentially downregulated genes affected by TsaB/YeaZ included the two-component system, starch and sucrose metabolism, pyruvate metabolism, PTS, pentose phosphate pathway, nitrogen metabolism, lysine degradation, glycolysis, glycolipid metabolism, fatty acid degradation, citrate cycle, carbon metabolism, arginine biosynthesis, arginine and proline metabolism, alanine, aspartate and glutamate metabolism, and ABC transporters (Figure 5C). In contrast, biosynthesis pathways of amino acids such as valine, leucine, and isoleucine biosynthesis, ribosome, pyrimidine metabolism, glycine, serine and threonine metabolism, C5-branched dibasic acid metabolism, 2-oxocarboxylic acid metabolism, and some ABC transporters were significantly upregulated among the 241 differentially upregulated genes by TsaB/YeaZ (Figure 5F).

**Figure 6:**
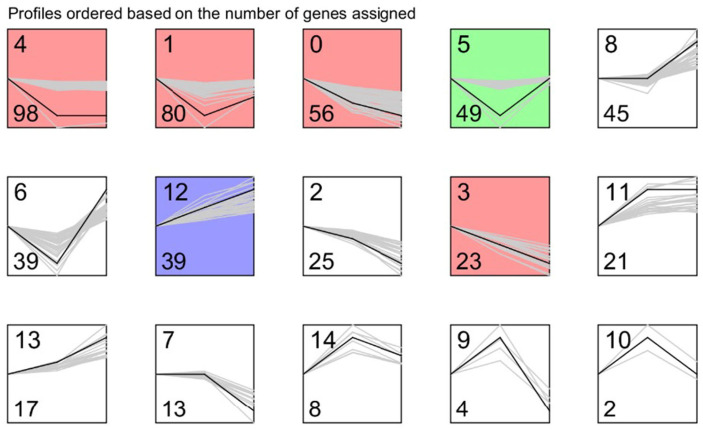
The interest trend class genes affected by TsaB/YeaZ at the early log, middle log, and early stationary phases of growth in TSB. The profiles are ordered based on the number of genes assigned. The top number represents the profile number, and the bottom number represents the gene number in each panel.

To further explore the differentially expressed genes during downregulating TsaB/YeaZ at different phases of growth, we determined the trend gene expression module using STEM software. We utilized the multiple of difference (logFC) of the differentially expressed genes in the OD 0.2, 0.5, 1.0 group for STEM trend analysis and identified 15 differential gene expression trend clustering modules (profiles 0 to 14), as shown in Figure 6 and Table 2. Furthermore, we conducted a pathway enrichment analysis of 160 genes belonging to the interest trend class I gene and revealed that the interest trend class I genes are mainly involved in the biosynthesis of secondary metabolites, microbial metabolism in different environments, ABC transporter pathways, glyceride metabolism, selenium compound metabolism, nitrogen metabolism, phosphoinositide metabolism, quorum sensing, and biosynthesis of amino acids (Table 3).

### 2.7. Identify Proteins That Potentially Interact with TsaB/YeaZ in S. aureus

**Figure 7:**
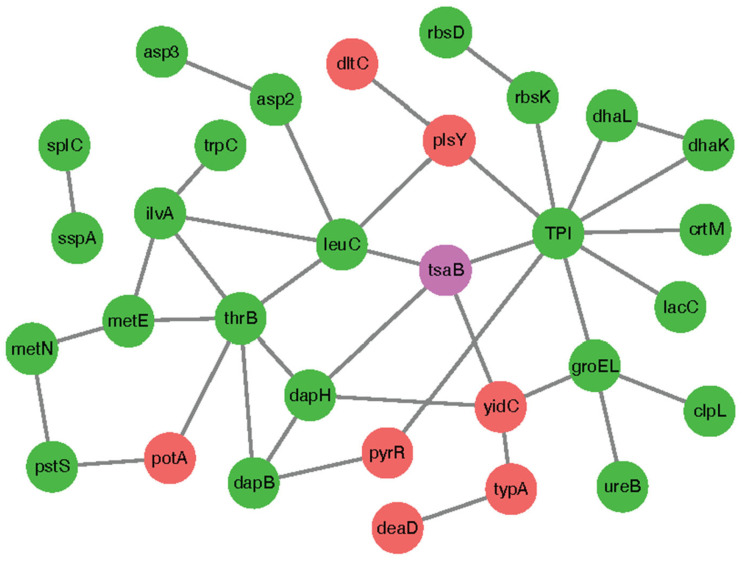
Protein-protein interaction network of TsaB/YeaZ for top 10 interest trend class I genes. Red dots represent the genes whose fold changes of differential expression are upregulated at OD600nm = 0.2, 0.5, and 1.0. Green dots represent the genes whose fold changes of differential expression are downregulated at OD600nm = 0.2, 0.5, and 1.0. Purple dots represent the *tsaB/yeaZ* gene.

To further explore the biological functions of TsaB/YeaZ, we performed protein and protein interaction (PPI) analysis of the TsaB/YeaZ gene and interest trend class I genes using the String11.5 database. A total of 34 nodes (node) and 40 edges (edge) were revealed in the TsaB/YeaZ network (Figure 7). Through the topological analysis of the network, we found that *tpi*, *thrB*, *leuC*, *dapH*, and other genes have a high degree of connection in the network (Table 4). These data suggest that those three proteins, TPI, LeuC, and DapH, also significantly interact with YeaZ/TsaB, which could serve as a potential hub gene. The TPI (E5491_RS04085, triose-phosphate isomerase) gene is directly involved in the pathways including ko01200 carbon metabolism, ko01230 biosynthesis of amino acids, ko00010 glycolysis/gluconeogenesis, ko00051 fructose and mannose metabolism, ko00562 Inositol phosphate metabolism, ko00710 carbon fixation in photosynthetic organisms, and other metabolism-related pathways.

## 3. Discussion

In this study, we have demonstrated that the promising antibacterial target protein TsaB/YeaZ plays important roles in the cell division and cell wall biosynthesis of *S. aureus*. Our transcriptomic results suggest that the transcriptional regulation of essential gene *dltC* and cell division regulator *gpsB* may contribute to the essential function of TsaB/YeaZ for bacterial survival and bacterial morphology [27,35]. MraZ is a transcriptional regulator involved in the growth and cell division of *E. coli*; however, MraZ has no impact on the growth of *S. aureus* MW2 [32] but plays a role in its pathogenicity [36], suggesting the altered *mraZ* transcription by the depletion of TsaB/YeaZ may not be associated with the essentiality of TsaB/YeaZ in *S. aureus* WCUH29. The change in bacterial cell wall structure might increase the resistance to detergent-induced bacterial autolysis and antibiotics-induced cell lysis [37]. Indeed, we revealed that TsaB/YeaZ controls the bacterial autolysis and cell wall recycling possibly through mediating the expression of CidA and LrgA, two key regulators of bacterial autolysis in *S. aureus* [29,30], as well as MurQ, a critical protein for bacterial peptidoglycan recycling in Gram-negative bacteria [28,38]. It was reported that the null mutation of MurQ could result in a dramatic disadvantage for *S. aureus* and *B. subtilis* survival in the late stationary phase growth [28]. Therefore, it is possible that the essentiality of TsaB/YeaZ for bacterial growth is partially attributed to its transcriptional regulation of MurQ.

The conserved essential protein TsaB/YeaZ is crucial for threonylcarbamoyl adenosine (t6A) biosynthesis in tRNA, influencing start codon selection and enhancing translation efficiency by preventing intramolecular base pairing. More than 70% of tRNAs, including tRNA-Ile, tRNA-Thr, tRNA-Ans, tRNA-Lys, tRNA-Ser, and tRNA-Arg, carry the t6A modification [9,10,12,31]. We have previously demonstrated the TsaB/YeaZ’s role in t6A biosynthesis in *S. aureus* [7]. Interestingly, our kinetic transcriptomic analysis also revealed the impact of TsaB/YeaZ on the transcription of these tRNAs during the early and middle log phases of growth (Tables S1 and S2), suggesting that TsaB/YeaZ may potentially impact translation dynamics. Taken together, these findings suggest that TsaB/YeaZ’s essentiality for bacterial growth may be attributed, at least in part, to its role in modulating tRNA transcription. By influencing the expression levels of tRNAs carrying the t6A modification, TsaB/YeaZ ensures the proper function of the translation machinery, thereby supporting essential cellular processes required for bacterial growth and survival. Therefore, the essentiality of TsaB/YeaZ for bacterial growth appears to be linked to its involvement in t6A biosynthesis, which in turn affects translation efficiency and may influence tRNA transcription dynamics. This highlights the multifaceted role of TsaB/YeaZ in ensuring the fidelity and efficiency of protein synthesis, crucial for bacterial viability and growth.

Our kinetic transcriptomic results showed that, in the absence of the inducer, IPTG obviously decreased the expression of TsaB/YeaZ in the early and middle log and early stationary phases of growth, indicating the specific downregulation of TsaB/YeaZ compared to the control strain. Moreover, the transcriptomic results indicate that TsaB/YeaZ negatively regulates the *ilv*-*leu* operon that is responsible for the biosynthesis of branch-chained amino acids (ILV), which is supported by both KEGG and GO BP enrichment pathway analyses (Figure 5 and Figure 6). These results are consistent with our previous finding that TsaB/YeaZ represses the transcription of the *ilv*-*leu* operon by directly binding to its promoter region [7], indicating the reliability of our transcriptomic analysis.

Furthermore, enrichment pathway analysis of interest trend class I genes, differentially expressed at various growth phases during TsaB/YeaZ depletion, suggests the involvement of TsaB/YeaZ in mediating quorum sensing, amino acid and secondary metabolite biosynthesis, ABC transporter pathways, and phosphoinositide metabolism, among others (Figure 5 and Figure 6). During the early log phase, the downregulation of pathways related to cellular signaling (two-component system, quorum sensing), energy metabolism (glycolysis, pyruvate metabolism), and protein synthesis (ribosome) suggests a reduction in cellular activity. Conversely, the upregulation of amino acid biosynthesis pathways indicates an increased demand for building blocks necessary for growth. In the middle log phase, similar to the early log phase, the downregulation of energy metabolism pathways persists, suggesting a slowing down of cellular processes. However, the upregulation of amino acid biosynthesis pathways continues, indicating the ongoing need for increased protein synthesis. In the early stationary phase, the downregulation of multiple metabolic pathways suggests a further slowdown of cellular metabolism. Nevertheless, the upregulation of amino acid biosynthesis pathways persists, indicating the continued need for protein synthesis even as growth slows. These findings collectively suggest that TsaB/YeaZ plays a pivotal role in modulating cellular metabolism to ensure the appropriate allocation of resources at different growth stages. This underscores its essentiality in regulating cellular homeostasis and adapting to varying environmental conditions.

The protein-protein interaction (PPI) analysis of TsaB/YeaZ, along with its interaction with proteins such as TPI, LeuC, and DapH, provides crucial insights into its essentiality mechanism. Observing significant interactions between TsaB/YeaZ and proteins such as TPI, LeuC, and DapH suggests that TsaB/YeaZ may serve as a potential hub gene in the cellular network, indicating its central role in coordinating interactions within essential metabolic pathways and cellular processes. For example, TPI is known to play a critical role in various metabolic pathways, including carbon metabolism, biosynthesis of amino acids, glycolysis/gluconeogenesis, and fructose and mannose metabolism [39]. These pathways are fundamental for cellular energy production and the synthesis of essential biomolecules required for bacterial growth and survival. The significance of these interactions is further highlighted by the high degree of connection observed in the network analysis. This reinforces the idea that TsaB/YeaZ plays a crucial role in mediating interactions within the cellular network involved in key metabolic processes. These findings suggest that TsaB/YeaZ’s essentiality for bacterial growth may be attributed, at least in part, to its central role in coordinating metabolic pathways critical for cellular function and survival. Disruption of its interactions with proteins such as TPI, LeuC, and DapH could potentially compromise essential cellular processes, thereby impacting bacterial viability and growth.

Furthermore, we observed that TsaB/YeaZ is involved in controlling the transcription of many virulence factors, including both those that are cell wall-associated, such as Protein A (*spa*), and numerous secreted toxins, such as *lukG*, *lukH*, *hly*, *hlgA*, *hlgB*, *hlgC*, PSM-alpha-2, and beta-class phenol-soluble modulin (Tables S1–S3) [40,41]. Downregulating the SrrAB two-component system by depleting TsaB/YeaZ may be involved in the regulation of virulence factors, because Protein A production was upregulated by the mutation of SrrB in microaerobic conditions, and SrrAB could act in the global regulation of virulence factors in *S. aureus* [33]. SrrAB is required for the transcription of *tst*, *spa*, and *icaR* under aerobic conditions, whereas SrrAB represses their transcription under low-oxygen conditions [34]. In addition, MraZ is a multi-functional regulator involved in diverse biological processes and can upregulate the expression of various virulence genes by *agr* and *sarA*. Therefore, it is possible that the regulation of virulence factors by TsaB/YeaZ is attributable to its regulation of the transcriptional regulator MraZ [32].

Moreover, our scanning and transmission electron microscopy results clearly indicate that TsaB/YeaZ negatively affects the morphology, specifically the size and smoothness, of *S. aureus*. It is unclear whether the over-transcription of *capABCDEFGLMN* operon responsible for the biosynthesis of capsule polysaccharide [42] is involved in the morphologic changes due to the downregulation of TsaB/YeaZ. The upregulation of single-strand binding proteins (*ssb*) by downregulating TsaB/YeaZ may also contribute to the cell size change, because overexpression of SSB could generate elongated cells [43]. However, conflicting findings regarding the influence of YgjD on bacterial morphologies have been documented. Handford et al. initially observed a mixed population of elongated and slightly enlarged short cells compared to the wildtype upon YgjD depletion [17], whereas Katz et al. noted shorter *E. coli* cells compared to the wildtype [44]. Bergmiller et al. conducted a single-cell time-lapse analysis on *E. coli* YgjD depletion mutants, revealing quantitative changes in the homeostasis of cell elongation and division rates across generations, resulting in reduced cell size and cessation of cell division [45]. This reduction in cell size mirrored morphological changes observed during the stringent response in *E. coli*, which is associated with the (p)ppGpp) [46,47,48]. The intracellular level of (p)ppGpp may account for the differing phenotypes observed in the two studies [17,44]. Katz et al. employed a relA + spot + strain, maintaining regular (p)ppGpp levels. In contrast, Handford et al. utilized MC4100, which carries a relA1 allele known to reduce (p)ppGpp levels under certain growth conditions [49].

Thus, one possible explanation is that the distinct phenotypes between the ruffled YgjD-depleted *E. coli* mutants [16] and the smooth TsaB/YeaZ-depleted *S. aureus* mutants are due to the nature of the strains with different (p)ppGpp levels or different impacts on the (p)ppGpp global signaling pathway between *E. coli* YgjD and *S. aureus* TsaB/YeaZ. Another possible explanation of the discrepancy is that YgjD and TsaB/YeaZ may possess different impacts on the capsule polysaccharide biosynthesis between Gram-negative *E. coli* and Gram-positive *S. aureus* cells.

Our previous studies have demonstrated that the essential TsaB/YeaZ protein binds its partner TsaD/Gcp, which is required for the essentiality of TsaG/Gcp for bacterial survival in *S. aureus* [14]. In this study, we found that TsaB/YeaZ possesses the similar functions of TsaD/Gcp controlling the bacterial autolysis by upregulating the expression of autolysis regulators CidA and LrgA [37], as well as repressing the branched-chain amino acids biosynthesis by downregulating the *ilv-leu* operon transcription in *S. aureus*, which is consistent with previous findings in *S. aureus* [13] and *E. coli* [50]. Therefore, it is possible the TsaB/YeaZ functions coordinately with TsaD/Gcp to regulate bacterial autolysis and the amino acids biosynthesis pathways.

It is well documented that branched-chain amino acids (BCAAs) play pivotal roles in *S. aureus* growth and virulence [51]. Consistent with our previous findings, kinetic transcriptomics has revealed that TsaB/YeaZ negatively regulates the expression of the *ilv*-*leu* operon, a regulation not solely linked to its necessity for growth [7]. These findings suggest that TsaB/YeaZ could potentially modulate the expression of virulence factors by influencing BCAA biosynthesis. In *S. aureus*, the *ilv*-*leu* operon encompasses *ilvDBHC-leuABCD-ilvA*, comprising 9 genes, akin to the arrangement in *B. subtilis* [52,53,54]. Despite possessing all the requisite genes for branched-chain amino acid, *S. aureus* demonstrates an auxotrophic phenotype for BCAAs, the underlying mechanism of which remains unidentified [55,56]. Various mechanisms governing the biosynthesis of BCAAs have been elucidated and characterized across different organisms [57,58,59].

In *S. aureus*, a global regulator, CodY, directly mediates the *ilv*-*leu* operon [57]. To elucidate whether TsaB/YeaZ indirectly affects the biosynthesis of amino acids and the expression of virulence factor via their transcriptional repressor CodY [57,60], we performed qPCR and found that the depletion of TsaB/YeaZ had no significant effect on the expression of CodY in *S. aureus*, which is consistent with the RNA-seq analysis and our previous finding [7]. These indicate that TsaB/YeaZ regulates the transcription of the *ilv-leu* operon in a CodY-independent manner. Meanwhile, we cannot rule out the possibility of Ile-tRNA-dependent regulation of *ilv-leu* operon because an Ile-T-box and a weak T-box motif are located in the upstream region of *ileS* encoding Ile-tRNA synthetase and the upstream region of *ilvDBHC-leuABCD-ilvA* operon, respectively, in *S. aureus* [61,62,63].

In conclusion, our findings indicate that TsaB/Yea Z is a multi-functional protein in *S. aureus* and may function as a global regulator to affect the cell division, cell wall biosynthesis, autolysis and peptidoglycan recycling, virulence factor, and biosynthesis of amino acids and capsule polysaccharide, as well as secondary metabolites, through direct transcription regulation and protein-protein interaction, or indirectly through mediating t6A biosynthesis in tRNA modification and/or expression. Targeting the essential functions of TsaR/YeaZ could present a promising avenue for the development of novel antibacterial agents to combat multidrug-resistant bacterial infections.

## 4. Materials and Methods

### 4.1. Bacterial Strains, Plasmids, and Growth Conditions

*S. aureus* WCUH29 is a methicillin-resistant clinical isolate (MRSA) [64]. The *S. aureus* control JW29011 (WCUH29 *attB*:: pLH1) and *Pspac*-regulated *yeaZ* expression mutant JW290211 (WCUH29 *∆yeaZ attB*:: *Pspac*-*yeaZ* containing plasmid pYH4-lacI) [7] were incubated in Tryptic soy broth (TSB) medium in the absence or presence of appropriate antibiotics (5 μg/mL erythromycin and 2.5 μg/mL tetracycline, Sigma-Aldrich, St. Louis, MO, USA) and inducer 100 μM of isopropyl β-D-1-thiogalactopyranoside (IPTG, Sigma-Aldrich, St. Louis, MO, USA) at 37 °C with shaking at 225 rpm.

### 4.2. Scanning Electron Microscopy (SEM)

Overnight cultures of *S. aureus* control strain JW29011 and the *Pspac*-regulated *yeaZ* expression mutant JW290211 were inoculated at 1% in fresh TSB medium, both in the absence and presence of 100 μM IPTG. Cells were harvested from the mid-logarithmic phase of growth (OD600nm reached 0.4–0.5). Subsequently, bacterial cells were fixed immediately in 2.5% Glutaraldehyde in 0.1 M sodium cacodylate buffer and left overnight at 4 °C. After fixation, cells were washed thrice with 0.1 M sodium cacodylate buffer and then post-fixed in 1% Osmium tetroxide in 0.1 M sodium cacodylate buffer, followed by three additional washes with distilled water. Dehydration was achieved using a gradient of 25–100% ethanol, followed by three changes of Hexamethyldisilane (HMDS) for 1 min each, and reconstitution with HMDS. Reconstituted samples were air-dried on coverslips, which were subsequently mounted on scanning electron microscopy stubs, coated with platinum using a sputter coater, and observed using a Hitachi S3500N scanning electron microscope (Hitachi High-Tech Corporation, Tokyo, Japan). Images were captured using Quartz PCI digital imaging software. Twenty bacterial cells were randomly selected from each sample, and the cell area was measured using iTem software.

### 4.3. Transmission Electron Microscopy (TEM)

Overnight cultures of *S. aureus* control strain JW29011 and the *Pspac*-regulated *yeaZ* expression mutant JW290211 were inoculated at 1% in fresh TSB medium, both in the absence and presence of 100 μM IPTG. Bacterial cells were harvested during the mid-logarithmic phase of growth and subjected to the same processing procedure as for SEM until dehydration using a 25–100% acetone gradient. Dehydrated samples were then infiltrated with a mixture of 2:1 Acetone: Embed 812 resin for 1 h, followed by a 1:2 Acetone:Embed 812 resin mixture for 1 h. Subsequently, they were infiltrated with 100% resin, embedded in gelatin capsules, and polymerized overnight at 58 °C. Embedded samples were trimmed and sectioned using a Leica UC6 Ultramicrotome. Thin sections (60–70 nm) were collected on a 200-mesh copper grid using a perfect loop and stained with 5% uranyl acetate and Sato lead citrate. These sections were observed under a JEOL 1200 EX II transmission electron microscope (Peabody, MA, USA), and images were captured using a Veleta 2K × 2K camera with iTem software. Twenty bacterial cells were randomly selected from each sample, and their area, perimeter, bacterial cell areas, and peptidoglycan thickness were measured using iTem software.

### 4.4. RNA Isolation and Purification

Overnight cultures of *S. aureus* control strain JW29011 and the *Pspac*-regulated *yeaZ* expression mutant JW290211 were inoculated at 1% in fresh TSB medium, both in the absence and presence of 100 μM IPTG, and were grown to early (OD600nm = 0.2) and mid-exponential (OD600nm = 0.5), and early stationary (OD600nm = 1.0) phases. Total RNA was extracted from these cultures using the SV total RNA isolation system (Promega, Z3100, Madison, WI, USA) or RiboPure™-Bacteria kits (Invitrogen, Thermo Fisher Scientific, Waltham, MA, USA) following standard protocols [65]. Briefly, bacterial cells were harvested by centrifugation at 4000× *g*, and the RNA was purified, followed by two rounds of DNase treatment (TURBO DNA-free kit, Ambion, Tempe, AZ, USA) to remove contaminating DNA. The concentration of RNA was determined spectrophotometrically at 260 nm.

### 4.5. RNA Sequencing (RNA-seq) and Data Analysis

#### 4.5.1. RNA Sequencing

Overnight cultures of *S. aureus* control JW29011 and *Pspac*-regulated *yeaZ* expression mutant JW290211were inoculated at 1% in fresh TSB medium in the absence and presence of 100 μM IPTG and grown to the early (OD_600nm_ = 0.2) and mid-exponential (OD_600nm_ = 0.5) and early stationary (OD_600nm_ = 1.0) phases. Total RNA was purified from the above cultures, as described [65]. The RNA sequencing analysis was performed as described [65]. Briefly, purified total RNA was processed by removing ribosomal RNAs using a Ribo-off rRNA depletion kit (bacteria), followed by cDNA synthesis and library construction using a VAHTS™ Stranded mRNA-seq Library Prep kit for Illumina^®^ (Vazyme Biotech Co., Ltd., Shanghai, China) and sequenced using the Illumina platform (Illumina, San Diego, CA, USA)

#### 4.5.2. Differential Gene Expression Analysis

Three independent biological repeats were included in each sample for RNA-seq analysis. Each mRNA sample included three independent biological repeats (*n* = 3), and we conducted DESeq analysis to identify genes whose transcriptions were significantly different with the condition of q Value ≤ 0.05 and log2 Fold Change ≥ 1. We calculated a total of 6 comparison groups, as shown in (Table 5).

#### 4.5.3. KEGG Pathway and Gene Ontology (GO) Biological Process (BP) Functional Enrichment Analysis

To identify biological pathways and functions that the essential protein TsaB/YeaZ is involved in *S. aureus*, we performed enrichment analysis of differentially expressed gene sets at different phases of growth using both the KEGG pathway and GO BP functional databases. To determine whether the differentially expressed genes are focused on certain functions, the R language cluster Profiler was used to analyze the functional enrichment of GO BP and KEGG pathways using Fisher’s exact test. The functional bubble diagram was generated by using the R ggplot2 package.

#### 4.5.4. Protein and Protein Interaction (PPI) Analysis of TsaB/YeaZ Gene and Interest Trend Genes Using the String 11.5 Database

*S. aureus* NCTC 8325 strain was used as a reference strain, and threshold combine score ≥ 0.4 indicates a significant relationship (PPI). PPI network diagrams were generated using the Cytoscape 3.6.1 software (https://cytoscape.org/) (accessed on 1 November 2022).

### 4.6. Semi-Quantitative Real-Time RT-PCR (qPCR) Analysis

To assess the impact of YeaZ on the expression of the *ilv*-*leu* operon, we utilized qPCR to compare RNA levels, following established procedures [65]. Overnight cultures of the *S. aureus* control strain JW29011 and the *Pspac*-regulated *yeaZ* expression mutant JW290211 were inoculated at 1% in fresh TSB medium, both in the absence and presence of 100 μM IPTG, and were grown to the mid-exponential phase (OD600nm = 0.5). Total RNA extraction was carried out from these cultures as previously described [65]. Subsequently, first-strand cDNA was synthesized using SuperScript III reverse transcriptase and random primers (Thermo Fisher Scientific, Waltham, MA, USA). Duplicate reverse transcription reactions were performed for each RNA sample, alongside a control reaction without reverse transcriptase. Additionally, PCR reactions were set up in duplicate to assess levels of DNA contamination. Real-time sequence-specific detection and relative quantitation were conducted using VeriQuest SYBR Green qPCR Master Mix (Thermo Fisher Scientific, Waltham, MA, USA) with the Stratagene Mx3000P Real-Time PCR System (Agilent Technologies, Santa Clara, CA, USA). Gene-specific primers designed to yield 100–200 bp specific products were utilized (Table 6) [7]. Relative quantification of the products was computed using the Comparative CT method as outlined for the Stratagene Mx3000P system, with the housekeeping gene 16S rRNA serving as an endogenous control.

### 4.7. Triton X-100-Induced Autolysis Assays

Autolytic assays were performed following our previously established protocol [37]. The *Pspac*-regulated *yeaZ* mutant and control strains were cultured overnight in TSB supplemented with 100 μM IPTG (isopropyl-β-d-thiogalactopyranoside), 5 μg/mL erythromycin (Erm), and 2.5 μg/mL tetracycline (Tc) at 37 °C with shaking at 220 rpm. Subsequently, the bacterial cultures were diluted 1:100 in fresh TSB containing 1 M NaCl, 5 μg/mL Erm, and 2.5 μg/mL Tc, with or without the inducer 100 μM IPTG, and incubated until reaching an OD580 of 0.5 to 0.6 at 37 °C with shaking. Bacterial cells were harvested by centrifugation at 4000× *g*, resuspended in an equal volume of buffer containing 50 mM Tris-HCl (pH 7.5) and 0.1% Triton X-100, and then incubated at 30 °C with shaking at 220 rpm. Changes in OD580 were measured every 30 min over a 4-h period. The results were normalized to the OD580 at time zero (OD0), representing percent lysis at time t = [(OD0 − OD at time t)/OD0] × 100. Each experiment was conducted independently at least three times.

## Figures and Tables

**Table 1:** Results of qPCR analysis during downregulating TsaB/YeaZ using *Pspac*-regulated *tsaB*/*yeaZ* expression strain.

Gene Name	Fold Change (Decrease) ^a^
*tsaB*/*yeaZ*	1.99 ± 0.52
*cidA*	1.88 ± 0.25
*murQ*	2.75 ± 0.48
*femA*	1.61 ± 0.19
*lytM*	1.97 ± 0.08
*lytN*	2.04 ± 0.31
*atl*	2.64 ± 0.46
*srrA*	3.28 ± 0.35
*hlgC*	3.06 ± 0.48
*lukH*	16.75 ± 2.32

^a^ The fold change represents the transcription levels of genes with the depletion of TsaB/YeaZ compared with those during the induction of *tsaB*/*yeaZ* transcription with IPTG (100 μM) at the exponential phase of growth (OD600nm ~ 0.5).

**Table 2:** The results of the Short Time-series Expression Miner (STEM) analysis.

Profile	Count	Trend	Class
0	56	Down-down	Interest trend class I gene
2	25	Down-down	
3	23	Down-down	
12	39	Up-up	
13	17	Up-up	
9	4	Up-down	Interest trend class II gene
10	2	Up-down	
14	8	Up-down	
1	80	Down-up	
5	49	Down-up	
6	39	Down-up	
4	98	Down-flat	Interest trend class III gene
11	21	Up-flat	
8	45	Flat-up	Interest trend class IV gene
7	13	Flat-down	

**Table 3:** KEGG enrichment pathways of interest trend class I genes.

Pathway ID	Description of Pathway	Count	*p*-Value
ko01110	Biosynthesis of secondary metabolites	21	5.55 × 10^−8^
ko01120	Microbial metabolism in diverse environments	14	8.07 × 10^−5^
ko02010	ABC transporters	16	0.004460232
ko00561	Glycerolipid metabolism	5	0.008120074
ko00450	Selenocompound metabolism	3	0.009847925
ko00910	Nitrogen metabolism	4	0.019201212
ko00562	Inositol phosphate metabolism	2	0.03031329
ko02024	Quorum sensing	8	0.043943233
ko01230	Biosynthesis of amino acids	14	0.044417679

**Table 4:** The top 10 interest trend class I genes involved in protein-protein interaction with TsaB/YeaZ in *S. aureus*.

Gene Name	Gene *orf* Number	Degree	Profile	Type
*tpi*	E5491_RS04085	9	3	down-down
*thrB*	E5491_RS06900	6	0	down-down
*leuC*	E5491_RS11545	5	0	down-down
*dapH*	E5491_RS07270	4	0	down-down
*tsaB/yeaZ*	E5491_RS11500	4		
*groEL*	E5491_RS11380	4	2	down-down
*yidC*	E5491_RS11755	4	13	up-up
*ilvA*	E5491_RS11555	4	0	down-down
*dapB*	E5491_RS07265	3	0	down-down
*metE*	E5491_RS01730	3	3	down-down
*plsY*	E5491_RS07035	3	13	up-up

**Table 5:** The results of differentially expressed genes (DEG) in different groups.

Group	IPTG –	IPTG +	DEG_Up	DEG_Down	Total
yeaZ mutant OD 0.2	yeaZ_02	yeaZ 02	292	98	390
yeaZ mutant OD 0.5	yeaZ_05	yeaZ 05	97	118	215
yeaZ mutant OD 1.0	yeaZ_10	yeaZ 10	241	126	367
Control OD 0.2	C_02	C02	2	3	5
Control OD 0.5	C_05	C05	0	0	0
Control OD 1.0	C_10	C10	0	0	0

*yeaZ* mutant: JW290211; Control: JW29011.

**Table 6:** Primers for qPCR analysis.

Primer Name	Oligo Sequences (5’-3’)
yeaZRTfor243	ACTGCTTGTCGTCTTGCATC
yeaZRTrev370	AACGCTAAAACATTAGCGTATGCGTTAG
cidA RT For	GTCTTTTTCTTCATACCGTCAGT
cidA RT Rev	TCATTCATAAGCGTCTACACCT
murQ RT For	GCTATGACGATGGCTGTAGAAG
murQ RT Rev	CACTCGCGGCAATTCCTATAA
femA RT For	TCATCGATTACAGACGAAGACAC
femA RT Rev	TCTTTTAGTTTAGACGGCGCAACC
lytM-F1	GCAGGAGATAACAATGACTACACAG
lytM-R1	GCTGTCGCTTTACTTGCTGAT
lytN RT For	AGCTGAACCTGGGGACTTAG
lytN RT Rev	CAACTTTATGTGCAACCTCTGC
atl RT For	GCTGGTTATAGTTTAGTTGATGATG
atl RT Rev	GGTTGTGCTGAAGCGCTAAAAG
srrAB-RT.2A	TGCCTGAAATGGATGGTATCC
srrAB-RT.2B	AACACGGTTTGTTTCTTCACC
hlgC RT For	CCCTCTTGCCAATCCGTTATTA
hlgC RT Rev	ATATCGCTTCCTTTACCGATGTC
lukH RT For	CCACTTCTTACTAATGCTGGGT
lukH RT Rev	TGACTTGAAGTATGGTGGAGAAG

## Data Availability

Data are contained within the article and supplementary materials.

## Supplementary Materials

The following supporting information can be downloaded at: https://www.mdpi.com/article/10.3390/antibiotics13050393/s1, Figure S1: GO enriched biological process pathways significantly affected by TsaB/YeaZ; Table S1: Differentially expressed genes of WCUH29 at early log phase by the depletion of TsaB/YeaZ; Table S2: Differentially expressed gens of WCUH29 at middle log phase by the depletion of TsaB/YeaZ; Table S3: Differentially expressed gens of WCUH29 at early stationary phase by the depletion of TsaB/YeaZ; Table S4: The genes that are overlapping up- and down-regulated by the depletion of TsaB/YeaZ at different growth phases of WCUH29; Table S5: DESeq analysis results at different growth phases of both *Pspac*-regulated *yeaZ* expression mutant and control strain.
