# Supplementary material for: New Insights into the Biological Functions of Essential TsaB/YeaZ Protein in Staphylococcus aureus
Source: Antibiotics (Basel). 2024 Apr 25;13(5):393. doi: 10.3390/antibiotics13050393 (PMC11117223; doi:10.3390/antibiotics13050393)
Supplement: Supplementary file 1 [file antibiotics-13-00393-s001.zip › Figure S1.pdf]

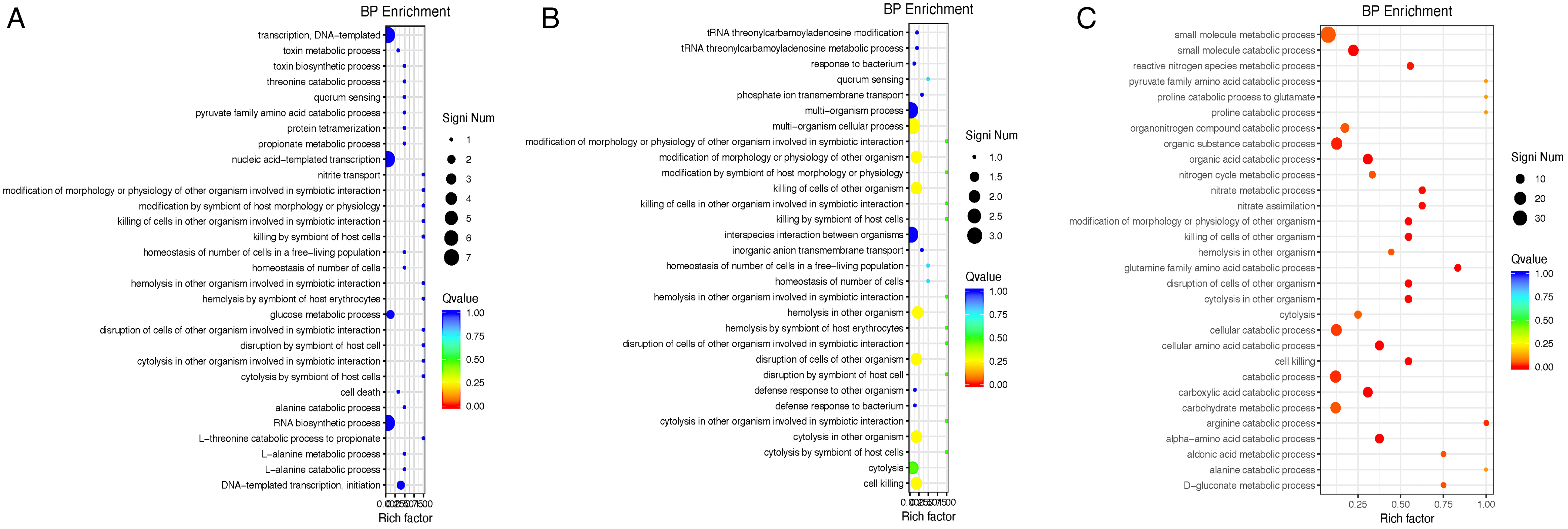

Figure S1. Downregulated enriched BP biological pathways A at OD 0.2, B at OD 0.5, and C at OD 1.0 after down-regulating TsaB/YeaZ.

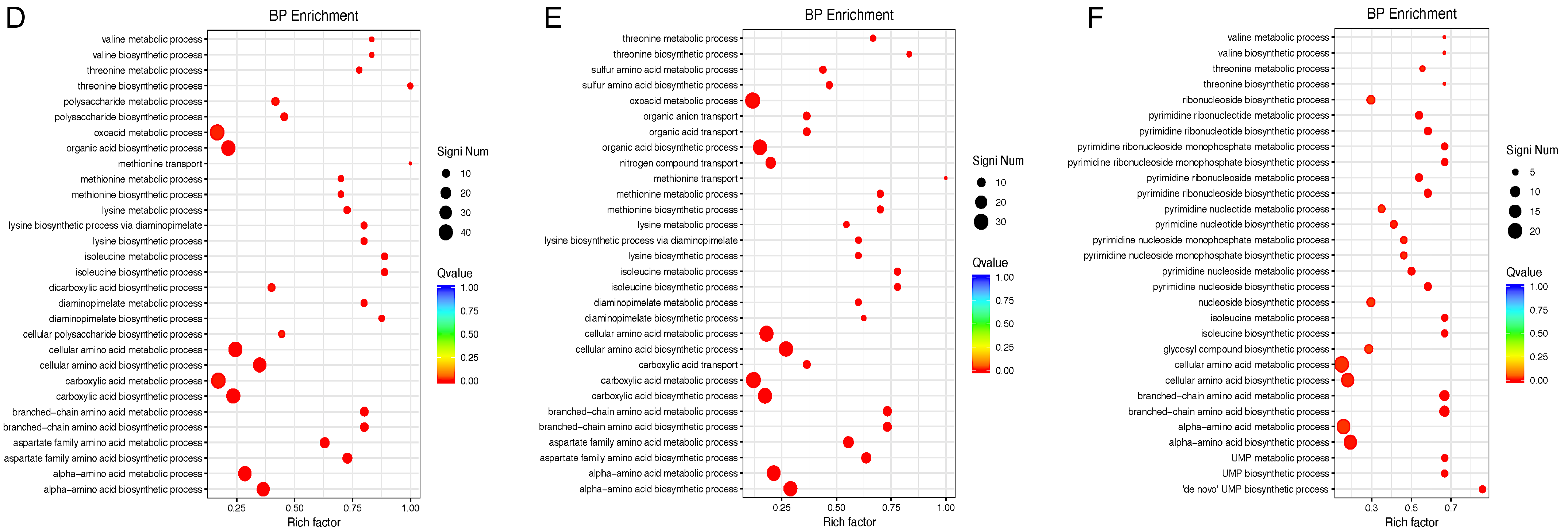

Figure S1. Upregulated enriched BP biological pathways D at OD 0.2, E at OD 0.5, and F at OD 1.0 after down-regulating TsaB/YeaZ.
